# Saharan Dust and Childhood Respiratory Symptoms in Benin

**DOI:** 10.3390/ijerph19084743

**Published:** 2022-04-14

**Authors:** Sara McElroy, Anna Dimitrova, Amato Evan, Tarik Benmarhnia

**Affiliations:** 1 Hebert Wertheim School of Public Health, University of California, La Jolla, CA 92093, USA; 2 San Diego State University, San Diego, CA 92182, USA; 3 Scripps Institution of Oceanography, University of California San Diego, La Jolla, CA 92093, USA; anna.k.dimitrova@gmail.com (A.D.); aevan@ucsd.edu (A.E.); tbenmarhnia@health.ucsd.edu (T.B.)

**Keywords:** dust, African dust, Saharan dust, respiratory outcomes, children under-5, low-to-middle-income countries

## Abstract

Mineral dust is one of the largest natural constituents of coarse particulate matter (PM_10_). Most of these dust emissions originate from northern Africa, and several hundred tera-grams of dust are emitted annually from this region. Previous evidence has linked dust PM_10_ to adverse respiratory outcomes in children. However, most of these studies have been from high-income countries (HICs) or examined dust from other regions of the world, mainly Asia. Evidence from low-to-middle-income countries (LMICs) in Africa is scarce. Respiratory infections are one of the leading causes of under-five mortality across the globe. However, there is a poignant disparity in studies examining these outcomes in children in the region where most dust is emitted. This study linked remotely sensed satellite data to a nationally representative survey to examine acute exposure to dust in children living in Benin using a time-stratified case-crossover analysis. We identified acute effects of exposure to dust and increased risk of cough in children under five. The effect of increased risk is strongest within two weeks of exposure and dissipates by four weeks. Children living in rural areas and households with lower income had a greater risk of adverse respiratory outcomes when exposed to dust. We could elucidate the specific period and conditions of increased risk for respiratory problems in children living in Benin.

## 1. Introduction

One of the largest natural contributors to particulate matter with a diameter less than 10 µm or less than 2.5 µm in length (PM_10_ and PM_2.5_) is mineral dust. The major dust sources emanate from arid regions in Africa, Asia, and the Middle East. Dust storms and plumes in these areas are the most prominent and extensive aerosol features visible in satellite images [1]. For example, several hundred tera-grams of dust are emitted each year from the Sahara desert to regions all over the globe [2]. Dust storms result from strong winds that disperse large amounts of desert dust into the air, resulting in extensive particulate exposure over large areas. These storms impact air quality on local and global scales and can lead to short- and long-term health effects [3,4,5]. Dust storms can generate high acute PM_10_ concentrations, which often exceed the World Health Organization’s (WHO) recommendations of safe levels (mean of 50 µm/m^3^ within 24 h) [6].

In the context of climate change, we have witnessed an increase in the frequency and magnitude of extreme weather events attributed to the rise in global surface temperatures and the associated change in precipitation regimes. These changes in weather patterns have also affected wind directions and the distribution of droughts, resulting in an overall decline of African dust [7,8]. Despite the observed reduction of North African dust, major dust storms still occur today and affect large geographical areas across Africa, the North Atlantic, and North America [9]. The largest source of dust emissions today is the Bodélé depression in Chad, dust from which travels over Northwest Africa [8,10] to countries such as Benin.

There is a consistent body of evidence that has linked PM with many adverse health outcomes. However, the majority of this evidence did not examine PM from dust. For example, studies have shown that short-term exposure to ambient PM (from any source) can increase the risk of cardiovascular and respiratory morbidity and mortality [11,12,13,14,15]. More specifically, short-term exposure to PM_10_ has been associated with an increased risk of cough and more severe respiratory infections in adults and children [16,17,18,19,20]. There are several proposed pathways through which dust can affect human health. African dust’s main constituents include clay, minerals (mainly iron), and quartz, but it can also contain microorganisms [21]. Aerosols rich in iron can cause pulmonary inflammation [22], while the presence of microorganisms can provoke immune responses [23]. Children constitute a particularly vulnerable group because they are more sensitive to air pollutants than adults [24].

Acute respiratory infections are among the leading cause of childhood mortality across the globe and accounted for 10% of deaths in children younger than five years in 2017 [25]. The respiratory infection burden varies significantly across the globe, disproportionately affecting the young and the poor [26]. People who have the highest risk of contracting or dying from respiratory infections are usually from rural households and with low income and education levels [27]. In this context, children residing in low-to-middle-income countries (LMICs) are one of the groups expected to be most vulnerable to such infections.

Most of the evidence examining how dust or PM_10_ attributable to dust affects respiratory symptoms in children has been derived from high-income countries or has focused on dust originating from Asia [28,29]. A recent systematic review examining the health effects of Asian dust identified only 12 out of 89 studies that focused primarily on child health [23]. There is even less evidence on the health effects of African dust on children in Sub-Saharan [30]. The few studies we identified on the topic found that dust storms increased child mortality in the West Africa region, which was most pronounced among poorer households [30,31]. We did not find studies that specifically looked at the impact of African dust storms on child respiratory health in Sub-Saharan Africa. This lack of evidence presents a major gap in the literature since acute respiratory infections, including pneumonia, are one of the region’s leading causes of child mortality. Moreover, repeated episodes of pneumonia can have long-lasting consequences for children’s respiratory health [32,33].

Additional evidence is urgently needed to address the high burden of respiratory health issues in children in LMICs through informing and implementing actionable policies. Korea provides one example of how targeted warnings can be implemented. The government has tried to reduce exposure to yellow dust; A warning system was developed with behavioral guidelines when PM_10_ levels exceed a certain threshold. These advisories have been linked to better health outcomes in children and higher birth weight of newborns [34].

When it comes to the health impacts of Saharan dust, the lack of evidence might be tied to the methodological challenges of conducting research in the region. Investigators must overcome two main challenges when estimating local health burdens from exposure to dust-related PM directly. The first challenge concerns measuring the exposure and health outcomes accurately. The second challenge concerns separating the impact of PM_10_ attributable to dust from other correlated variables that might also influence children’s health, such as economic activities such as industrial production. This study will overcome these challenges by utilizing remotely sensed Moderate Resolution Imaging Spectroradiometer (MODIS) data from NASA’s Aqua and Terra satellites and linking these data with the Demographic Health Survey (DHS), a georeferenced household survey.

We implement a case study in Benin examining the link between acute exposure to dust and childhood cough. We chose Benin for our case study because the country is an area that frequently experiences large dust events from the Sahara-Sahel region. Specifically, dust emissions from the Bodélé depression have been shown to disperse over Benin. A study done by Tulet comparing satellite images of dust storms and simulated data found that the majority of dust emitted from the massive dust storm in March of 2006 was concentrated in Benin [35]. Another study examining the composition of atmospheric aerosols in Benin found that dust made up 26–59% of the total aerosols [36]. Benin is thus considered to be an adequate location for this case-study.

## 2. Materials and Methods

### 2.1. Health Data

Data for children under five years were obtained from the DHS database [37]. DHS surveys are nationally representative and focus on fertility behavior, health, and wellbeing of women of reproductive age and their children. We used the most recent Benin’s DHS phase 7 survey (November 2017–February 2018). Women aged 15–49 responded to questionnaires about their complete birth histories and provided detailed health information about their children. In addition, individual- and household-level data, such as household relative wealth status, educational level of household members, prenatal care, and health insurance status, are recorded. DHS surveys also include global positioning system (GPS) data for each primary sampling unit (PSU). A PSU is defined as a city block in an urban area and a village in a rural area. Surveyors used global positioning system devices to collect geospatial information to identify the central point of the populated area of each PSU [38]. In DHS surveys, GPS coordinates are randomly displaced to ensure respondent confidentiality. The displacement is carried out so that urban PSUs contain a minimum of 0 and a maximum of 2 km of error, and rural PSUs contain a minimum of 0 and a maximum of 5 km of positional error with a further 1% of the rural clusters displaced by a minimum of 9 and a maximum of 10 km [39].

### 2.2. Exposure Data and Classification

A MODIS Terra and Aqua combined Multi-angle Implementation of Atmosphere Correction (MAIAC) Land Aerosol Optical Depth (AOD) data product was utilized to determine if there was a presence of dust in the air around the time of the survey data collections. This product is a gridded Level 2 with a spatial resolution of 1 km and a temporal resolution of 1 day (MCD19A2.006). The Optical_Depth_047 band was downloaded using Google Earth Engine (GEE). The daily optical depth was obtained from 1 November 2017 to 28 February 2018, for each 1-km pixel within Benin. In addition to the AOD measurements, there is a band called AOD_QA, a 16-bit unsigned integer. Each grouping of bits indicates certain information. Bits 0–2 reveal information about cloud cover, bits 3–4 contain information about the land, water, snow, or ice mask, bits 5–7 tell how close the pixel is to a cloudy pixel, bits 8–11 are quality assurance measures for AOD, bit 12 shows if there is a glint, and bits 13–14 indicate if there is the detection of smoke or dust [40]. The dust model produced a unique 16-digit number for the AOD_QA band and was downloaded for each PSU in Benin.

**Figure 1:**
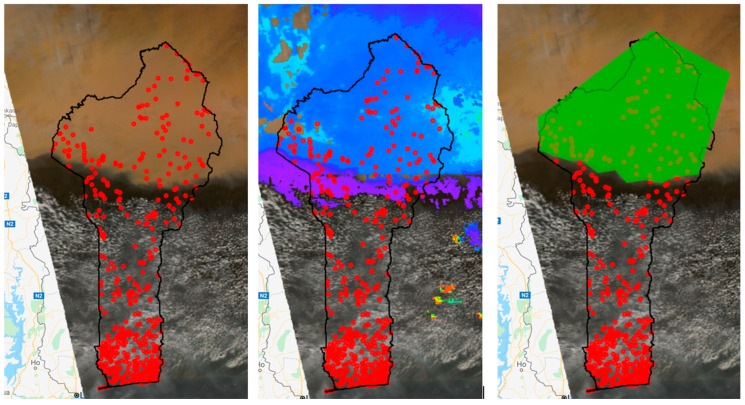
Satellite image of Benin with locations of each PSU (red circles). True-color image of a dust storm in Benin using the MYD09GA.006 Aqua Surface Reflectance Daily Global 1 km and 500 km resolution product (**left**). The same image with the Terra and Aqua MAIAC Land Aerosol Optical Depth 1 km added as a layer (showing the dust storm in blue, **center**). The same image with the manually drawn polygon around the dust cloud and indicating the exposed PSUs within the polygon (**right**).

For each day in the study period (*n* = 73), the presence of dust was visually inspected for Benin. True-color images (red, green, and blue bands) were visually inspected using the MODIS Surface Reflectance product for the presence of dust. Each day within the study period, this product was filtered, and exposure ascertainment was determined. When dust was observed, a polygon was drawn around the dust plume. Any PSUs within this polygon were considered exposed, and any PSUs located outside the polygon were classified as unexposed (Figure 1). If the presence of dust could not be visually ascertained because of cloud cover or unclear images, the measure of the AOD_QA band was used to classify exposure status. This measure was also used to validate the visual classifications. The exposure data and health data were linked using the GPS location of PSUs, resulting in a binary exposure classification for each day per PSU.

### 2.3. Outcome Classification

Each mother was asked whether their children under five experienced cough symptoms within the two weeks preceding the interview date. If such symptoms were reported, the mother was further asked whether the child experienced short and rapid breathing and whether the child had a problem in the chest. If the child had a cough with short and rapid breathing associated with chest problems, the child was considered to be suffering from an acute respiratory infection (ARI).

### 2.4. Statistical Analyses

A time-stratified case-crossover analysis was implemented to examine the acute effects of dust exposure on childhood cough. This approach has been well established in the literature to estimate acute health effects of environmental exposure. It has been widely applied to estimate associations between short-term exposure to air pollution and health outcomes [41,42,43,44,45]. The outcome of cough (and ARI) is the unit of observation or case day, and dust data on the date of the outcome event is compared with three control days. For the purpose of this study, we match the case days to control days that are the same day of the week, in the same month and year as the case day. The matching on day of the week controls for potential week-varying confounders such as the week/weekend difference in environmental factors. Matching by month and year controls for potential confounding by seasonality and long-term trends [46].

We conducted two analyses that examined acute effects at different time periods. The first analysis entailed the case date to be defined as the day two-weeks prior to the interview date. Control days were then matched to this case date. An additional analysis was implemented, with the case date being defined as the date four weeks prior to the interview date. We chose this timeframe to gain insights into any lagged effects. Conditional logistic regression models were run for each of these two time periods. Time-variant confounders such as meteorological data were controlled for in the analyses. The land surface temperature was measured using the MODIS Land Surface Temperature and Emissivity product and was adjusted for in the regression models.

### 2.5. Effect Measure Modification

We explored the role of effect measure modifiers by stratifying the regression models by urban or rural residence, level of maternal education, rudimentary or finished housing materials, and household wealth. The DHS survey generates a wealth index, and it is a composite measure of a household’s cumulative living standard. Variables included in calculating the wealth index include household assets, building material, and types of water and sanitation facilities [47]. Maternal education is a continuous measure of the number of years of total education the mother had. A binary variable was created from this continuous measure to indicate if the mother received more education or zero to little education. A Cochran’s Q test of heterogeneity was performed to determine if there were differences between the stratified groups.

## 3. Results

Benin’s DHS phase 7 survey recorded information on 13,589 children. There was a total of 2018 cases of cough reported from 23 November 2017 to 4 February 2018. The majority of households in Benin lived in rural areas, had the lowest household wealth index measure, and the mean years of maternal education were about three years (Table 1).

**Figure 2:**
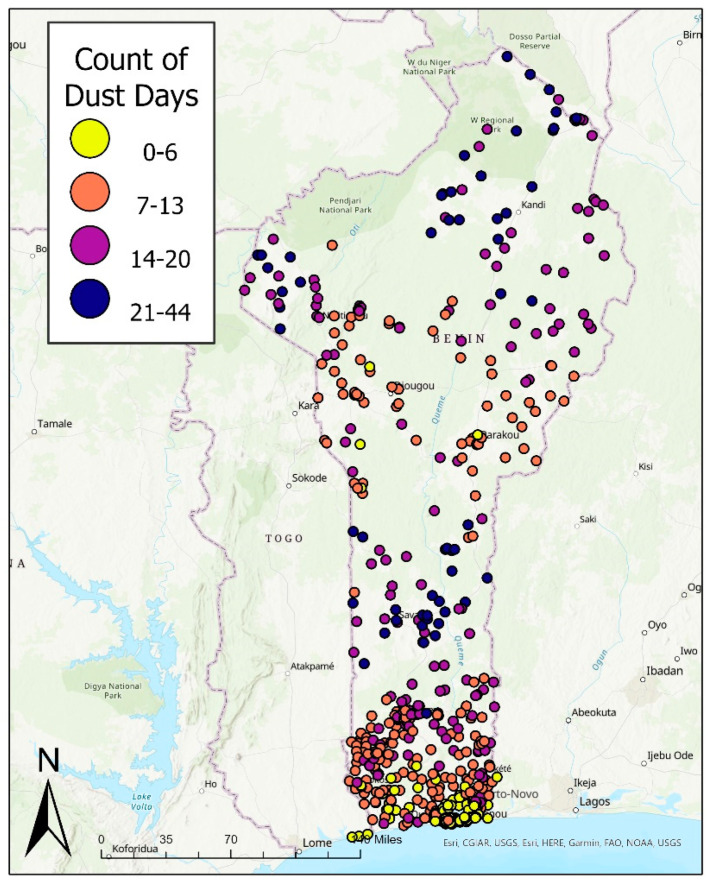
Total number of exposed days to dust per PSU in Benin from 23 November 2017 to 4 February 2018.

There was dust found in about 10% of the case dates (two weeks prior to the interview date). When examining the time period of four weeks prior to the interview, about 8% of case days were classified as exposed to dust. Figure 2 illustrates the total number of days (between 23 November 2017, and 4 February 2018) where dust was detected for each PSU location. The areas with the highest number of dust days were in northern and central Benin. Southern Benin experienced the lowest number of dust days throughout the study period.

**Figure 3:**
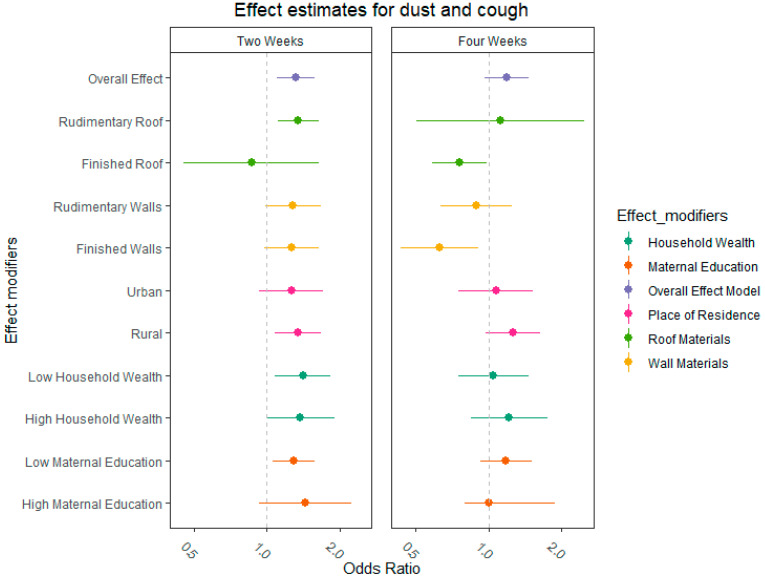
Odds ratios of overall effect model and models examining effect measure modification for both two- and four-weeks periods prior to the interview date. Each color represents a different effect modifier.

Figure 3 depicts the odds ratios for the two- and four-week periods. We observed an increased risk of cough within the two weeks prior to the interview when a child was exposed to dust (OR: 1.32, 95% CIs: 1.11–1.56). The Cochran Q heterogeneity found no significant differences in the effect modifiers (Appendix A), but there seems to be a general pattern of effect differences. Households with finished wall material were more protected from dust than households with rudimentary wall materials. Moreover, households with finished roofing decreased the risk of cough in children living in them. Children living in rural areas seem to have a higher risk of cough due to dust exposure than children living in urban locations. Similarly, children born in poorer households (1st and 2nd wealth quantiles) seem to be at an increased risk of cough after experiencing a dust storm compared to children from more affluent households; however, this difference is only observed for the two-week exposure period. Mothers’ level of education does not seem to moderate their children’s risk of cough due to dust exposure. Overall, we found inconclusive evidence for longer-term effects (up to 4 weeks) of exposure to dust on the risk of cough.

## 4. Discussion

Our study is one of a few studies to examine how acute exposure to African dust storms can affect children’s health in LMICs in Sub-Saharan Africa. Our analyses identified acute effects of increased risk of cough in children under five who lived in Benin and were exposed to dust storms. In addition, we found disparities in the risk of cough depending on the family’s relative wealth status and type of place of residence (rural or urban). Children living in poorer, rural conditions seem to be especially susceptible to the risk of cough due to dust storms compared to children living in urban areas and with wealthier households. One explanation for this urban-rural divide could be the generally higher frequency of dust storms in the northern part of the country, which is predominantly rural and sparsely populated. People residing in urban areas may also have better access to information about approaching dust storms, leading to improved behavioral responses.

One major advantage of our study is utilizing remote sensing data to classify dust exposure. Previous evidence has relied on ground monitors, which can be unreliable when measuring dust. Weather stations measure visibility and can therefore only capture the presence of visible dust in the air, but these measures are inadequate and not always reported [30,48]. These stations are also geographically sparse across Benin, which calls for the application of satellite-based dust observations. By utilizing NASA’s daily MAIAC aerosol optical depth and land surfacer reflectance products, we were able to study the acute effects of dust exposure on childhood respiratory health, using symptoms of cough as a proxy for respiratory health issues. Much of the previous literature has examined the association between dust and respiratory health issues on a monthly or annual scale, which is insufficient to capture the short-term and acute effects of dust exposure on children. Indeed, our results show that the effect diminished beyond two weeks of symptomatic illness. This study also examined the spatiotemporal distribution of dust events that can influence childhood cough across Benin. By linking these remotely sensed data with the DHS survey PSU locations, we were able to identify specific areas within Benin where the risk of Saharan dust storms is the highest. This information could be used to develop actionable policies in implementing early warning systems.

Identifying the effects of dust on child health presents a complex set of challenges. One primary concern is that high dust levels can be correlated with drought and soil conditions that may independently influence a child’s health status through mechanisms other than exposure to PM. Water scarcity and dry soil conditions can influence agriculture and economic activities, among other factors, which can affect children’s health indirectly (e.g., through malnutrition) or directly (e.g., through the consumption of unsafe drinking water) [49,50,51,52]. To account for these correlations, Benin was chosen as a case study due to the fact that the majority of dust over Benin originates from the Bodélé depression and is transported over a long-range distance [8,10]. Thus, it is reasonable to assume that the drought and soil conditions that created the right environment for significant dust emission episodes in the Bodélé region are far from the source of the health data. The study design inherently controls for these conditions at the local level, and land surface temperature was also included in the regression models as a possible time-varying confounder. Additionally, the cough outcome is self-reported and not diagnosed by a medical professional, which could lead to outcome misclassification. We also do not know when the cough occurred (i.e., before or after the dust event). Another limitation of this study is possible exposure misclassification. By visually examining satellite images for dust, there can be human error. Clouds, smoke, and dust may appear similar on satellite images. However, the exposure classification accuracy in this study was greatly improved with the addition of the AOD dust model data.

Moreover, such possible outcome or exposure misclassification is expected to result in estimates closer to the null, or more conservative than the true estimate. However, we were able to identify positive and statistically significant effect sizes, which indicates a true association. Our results can be considered conservative since factors such as concentration of dust in the air and duration of exposure were not accounted for in the analyses. An additional limitation that we acknowledge involves the selective survival of children. The most severely affected children may not have survived to the time of the interview, in which case our results would be biased downwards. Finally, due to the cross-sectional nature of the DHS surveys, we were not able to study these trends across time.

## 5. Conclusions

Even though dust emitted from the Bodélé depression region is predicted to decrease in the context of climate change, other areas of dust emissions are predicted to increase, mainly in the United States and southern Africa [53,54]. These new patterns in dust emissions will create new areas of increased vulnerability to dust. Given the above, it is imperative that we better understand the dangers of dust emissions and transport on human health, particularly for young children who are very sensitive to such air pollutants. Information from this study could be used to inform and target early warning systems in Benin.

## Figures and Tables

**Table 1:** Descriptive statistics of the study population of households of children under 5 in Benin.

	Overall (N = 13,589)
**Outcome Status**	
Cough	2018 (14.9%)
No Cough	10,520 (77.4%)
Missing	1051 (7.7%)
**Exposure Status**	
Dust present	1314 (9.7%)
Dust not present	12,132 (89.3%)
Missing	143 (1.1%)
**Household Wealth**	
1-Low wealth	3020 (22.2%)
2	2776 (20.4%)
3	2670 (19.6%)
4	2639 (19.4%)
5-High wealth	2484 (18.3%)
**Maternal Education**	
Mean (SD)	2.18 (3.65)
Median [Min, Max]	0 [0, 17.0]
**Place of Residence**	
Urban	5401 (39.7%)
Rural	8188 (60.3%)

## Data Availability

Data supporting reported results can be found at https://dhsprogram.com/Data/ (accessed on 1 March 2022).

## Appendix A

**Table:**

	Two Weeks		Four Weeks	
Effect Modifier	Cochran Q Estimate	χ^2^	Cochran Q Estimate	χ^2^
Urban	0.103	0.748	0.522	0.47
Rural				
High education	0.187	0.665	0.303	0.582
Low education				
High Household wealth	0.327	0.568	0.578	0.447
Low Household wealth				

## References

[1] Prospero J.M., Lamb P.J. (2003). African Droughts and Dust Transport to the Caribbean: Climate Change Implications. Science.

[2] Samoli E., Kougea E., Kassomenos P., Analitis A., Katsouyanni K. (2011). Does the presence of desert dust modify the effect of PM10 on mortality in Athens, Greece?. Sci. Total Environ..

[3] Cook A., Weinstein P., Centeno J. (2005). Health effects of natural dust. Biol. Trace Elem. Res..

[4] De Longueville F., Ozer P., Doumbia S., Henry S. (2013). Desert dust impacts on human health: An alarming worldwide reality and a need for studies in West Africa. Int. J. Biometeorol..

[5] Kotsyfakis M., Zarogiannis S.G., Patelarou E., Sarogiannis S. (2019). The health impact of Saharan dust exposure. Int. J. Occup. Med. Environ. Health.

[6] Leys J.F., Heidenreich S.K., Strong C.L., McTainsh G.H., Quigley S. (2011). PM10 concentrations and mass transport during “Red Dawn”—Sydney 23 September 2009. Aeolian Res..

[7] Evan A.T., Flamant C., Gaetani M., Guichard F. (2016). The past, present and future of African dust. Nature.

[8] Evan A.T., Fiedler S., Zhao C., Menut L., Schepanski K., Flamant C., Doherty O. (2015). Derivation of an observation-based map of North African dust emission. Aeolian Res..

[9] Francis D., Fonseca R., Nelli N., Cuesta J., Weston M., Evan A., Temimi M. (2020). The Atmospheric Drivers of the Major Saharan Dust Storm in June 2020. Geophys. Res. Lett..

[10] Koren I., Kaufman Y.J., Washington R., Todd M.C., Rudich Y., Martins J.V., Rosenfeld D. (2006). The Bodélé depression: A single spot in the Sahara that provides most of the mineral dust to the Amazon forest. Environ. Res. Lett..

[11] Analitis A., Katsouyanni K., Dimakopoulou K., Samoli E., Nikoloulopoulos A.K., Petasakis Y., Touloumi G., Schwartz J., Anderson H.R., Cambra K. (2006). Short-Term Effects of Ambient Particles on Cardiovascular and Respiratory Mortality. Epidemiology.

[12] Anderson H.R., Atkinson R.W., Peacock J., Marston L., Konstantinou K., WHO Organization (2004). Meta-Analysis of Time-Series Studies and Panel Studies of Particulate Matter (PM) and Ozone (O3): Report of a WHO Task Group.

[13] Anderson H.R., Atkinson R.W., Peacock J.L., Sweeting M.J., Marston L. (2005). Ambient particulate matter and health effects: Publication bias in studies of short-term associations. Epidemiology.

[14] Pope C.A., Dockery D.W. (2006). Health Effects of Fine Particulate Air Pollution: Lines that Connect. J. Air Waste Manag. Assoc..

[15] Xing Y.-F., Xu Y.-H., Shi M.-H., Lian Y.-X. (2016). The impact of PM2.5 on the human respiratory system. J. Thorac. Dis..

[16] Schindler C., Keidel D., Gerbase M.W., Zemp E., Bettschart R., Brändli O., Brutsche M., Burdet L., Karrer W., Knöpfli B. (2009). Improvements in PM10Exposure and Reduced Rates of Respiratory Symptoms in a Cohort of Swiss Adults (SAPALDIA). Am. J. Respir. Crit. Care Med..

[17] Zemp E., Elsasser S., Schindler C., Künzli N., Perruchoud A.P., Domenighetti G., Medici T., Ackermann-Liebrich U., Leuenberger P., Monn C. (1999). Long-Term Ambient Air Pollution and Respiratory Symptoms in Adults (SAPALDIA Study). Am. J. Respir. Crit. Care Med..

[18] Were F.H., Wafula G.A., Lukorito C.B., Kamanu T.K. (2020). Levels of PM10 and PM2.5 and Respiratory Health Impacts on School-Going Children in Kenya. J. Health Pollut..

[19] Nascimento A.P., Santos J.M., Mill J.G., Toledo de Almeida Albuquerque T., Costa Reis Júnior N., Reisen V.A., Coelho Pagel E. (2020). Association between the incidence of acute respiratory diseases in children and ambient concentrations of SO2, PM10 and chemical elements in fine particles. Environ. Res..

[20] Fan X.-J., Yang C., Zhang L., Fan Q., Li T., Bai X., Zhao Z.-H., Zhang X., Norback D. (2017). Asthma symptoms among Chinese children: The role of ventilation and PM10 exposure at school and home. Int. J. Tuberc. Lung Dis..

[21] Gyan K., Henry W., Lacaille S., Laloo A., Lamsee-Ebanks C., McKay S., Antoine R.M., Monteil M.A. (2005). African dust clouds are associated with increased paediatric asthma accident and emergency admissions on the Caribbean island of Trinidad. Int. J. Biometeorol..

[22] Prospero J.M. (1999). Long-term measurements of the transport of African mineral dust to the southeastern United States: Impli-cations for regional air quality. J. Geophys. Res. Atmos..

[23] Hashizume M., Kim Y., Ng C.F.S., Chung Y., Madaniyazi L., Bell M.L., Guo Y.L., Kan H., Honda Y., Yi S.-M. (2020). Health Effects of Asian Dust: A Systematic Review and Meta-Analysis. Environ. Health Perspect..

[24] Hasunuma H., Takeuchi A., Ono R., Amimoto Y., Hwang Y.H., Uno I., Shimizu A., Nishiwaki Y., Hashizume M., Askew D. (2021). Effect of Asian dust on respiratory symptoms among children with and without asthma, and their sen-sitivity. Sci. Total Environ..

[25] WHO Causes of Child Mortality. https://www.who.int/gho/child_health/mortality/causes/en/.

[26] UNICEF (2016). One is Too Many: Ending Child Deaths from Pneumonia and Diarrhoea.

[27] UNICEF, WHO (2013). End Preventable Deaths: Global Action Plan for Prevention and Control of Pneumonia and Diarrhoea.

[28] Alessandrini E.R., Stafoggia M., Faustini A., Gobbi G.P., Forastiere F. (2013). Saharan dust and the association between particulate matter and daily hospitalisations in Rome, Italy. Occup. Environ. Med..

[29] Kanatani K.T., Ito I., Al-Delaimy W., Adachi Y., Mathews W.C., Ramsdell J.W. (2010). Desert Dust Exposure Is Associated with Increased Risk of Asthma Hospitalization in Children. Am. J. Respir. Crit. Care Med..

[30] Foreman T. (2018). The Effect of Dust Storms on Child Health in West Africa.

[31] Adhvaryu A., Bharadwaj P., Fenske J., Nyshadham A., Stanley R. (2019). Dust and Death: Evidence from the West African Harmattan.

[32] Edmond K., Scott S., Korczak V., Ward C., Sanderson C., Theodoratou E., Clark A., Griffiths U., Rudan I., Campbell H. (2012). Long Term Sequelae from Childhood Pneumonia; Systematic Review and Meta-Analysis. PLoS ONE.

[33] Grimwood K., Chang A.B. (2015). Long-term effects of pneumonia in young children. Pneumonia.

[34] Baek D., Altindag D., Mocan N. (2017). Chinese Yellow Dust and Korean Infant Health. Soc. Sci. Med..

[35] Tulet P., Mallet M., Pont V., Pelon J., Boone A. (2008). The 7–13 March 2006 dust storm over West Africa: Generation, transport, and vertical stratification. J. Geophys. Res. Earth Surf..

[36] Ouafo-Leumbe M.-R., Galy-Lacaux C., Liousse C., Pont V., Akpo A., Doumbia T., Gardrat E., Zouiten C., Sigha-Nkamdjou L., Ekodeck G.E. (2018). Chemical composition and sources of atmospheric aerosols at Djougou (Benin). Arch. Meteorol. Geophys. Bioclimatol. Ser. B.

[37] ICF (2020). Demographic and Health Surveys (Various).

[38] Aliaga A., Ren R. (2006). The Optimal Sample Sizes for Two-Stage Cluster Sampling in Demographic and Health Surveys.

[39] USAID (2013). Geographic Displacement Procedure and Georefferenced Data Relaese Policy for the Demographic and Health Surveys. DHS. https://dhsprogram.com/pubs/pdf/SAR7/SAR7.pdf.

[40] Lyapustin A. MODIS Multi-Angle Implementation of Atmospheric Correction (MAIAC) Data User’s Guide. NASA 2018, Collection 6. https://lpdaac.usgs.gov/documents/110/MCD19_User_Guide_V6.pdf.

[41] Kloog I., Zanobetti A., Nordio F., Coull B.A., Baccarelli A.A., Schwartz J. (2015). Effects of airborne fine particles (PM 2.5) on deep vein thrombosis admissions in the northeastern United States. J. Thromb. Haemost..

[42] Wellenius G.A., Burger M.R., Coull B.A., Schwartz J., Suh H.H., Koutrakis P., Schlaug G., Gold D.R., Mittleman M.A. (2012). Ambient Air Pollution and the Risk of Acute Ischemic Stroke. Arch. Intern. Med..

[43] Zanobetti A., Dominici F., Wang Y., Schwartz J.D. (2014). A national case-crossover analysis of the short-term effect of PM2.5 on hospitalizations and mortality in subjects with diabetes and neurological disorders. Environ. Health.

[44] Di Q., Dai L., Wang Y., Zanobetti A., Choirat C., Schwartz J.D., Dominici F. (2017). Association of Short-term Exposure to Air Pollution with Mortality in Older Adults. JAMA J. Am. Med Assoc..

[45] Nhung N.T.T., Amini H., Schindler C., Joss M.K., Dien T.M., Probst-Hensch N., Perez L., Künzli N. (2017). Short-term association between ambient air pollution and pneumonia in children: A systematic review and meta-analysis of time-series and case-crossover studies. Environ. Pollut..

[46] Navidi W. (1998). Bidirectional case-crossover designs for exposures with time trends. Biometrics.

[47] Program D. (2020). Wealth Index Construction. https://dhsprogram.com/topics/wealth-index/index.cfm.

[48] Tong D.Q., Dan M., Wang T., Lee P. (2012). Long-term dust climatology in the western United States reconstructed from routine aerosol ground monitoring. Atmos. Chem. Phys..

[49] Few R. (2007). Health and climatic hazards: Framing social research on vulnerability, response and adaptation. Glob. Environ. Chang..

[50] Haines A., Kovats R.S., Campbell-Lendrum D., Corvalán C. (2006). Climate change and human health: Impacts, vulnerability, and mitigation. Lancet.

[51] Walson J.L., Berkley J.A. (2018). The impact of malnutrition on childhood infections. Curr. Opin. Infect. Dis..

[52] Levy K., Woster A.P., Goldstein R.S., Carlton E.J. (2016). Untangling the impacts of climate change on waterborne diseases: A systematic review of relationships between diarrheal diseases and temperature, rainfall, flooding, and drought. Environ. Sci. Technol..

[53] Pu B., Ginoux P. (2017). Projection of American dustiness in the late 21st century due to climate change. Sci. Rep..

[54] Bhattachan A., D’Odorico P., Dintwe K., Okin G.S., Collins S.L. (2014). Resilience and recovery potential of duneland vegetation in the southern Kalahari. Ecosphere.

